# A novel automated CT biomarker to predict outcomes in acute ischemic stroke: net water uptake

**DOI:** 10.3389/fneur.2025.1629434

**Published:** 2025-08-22

**Authors:** Monica Mallavarapu, Hyun Woo Kim, Ananya Iyyangar, Sergio Salazar-Marioni, Albert J. Yoo, Luca Giancardo, Sunil A. Sheth, Jerome A. Jeevarajan

**Affiliations:** ^1^McGovern Medical School, Department of Neurology, The University of Texas Health Houston, Houston, TX, United States; ^2^Texas Stroke Institute, Plano, TX, United States

**Keywords:** net water uptake, ischemic stroke, computed tomography, aspects, neuroimaging

## Abstract

**Background:**

Recent trials of large core thrombectomy have shown that our traditional understanding of infarct characteristics and reperfusion benefit may be incomplete for patients with acute ischemic stroke (AIS). The Alberta Stroke Program Early CT Score (ASPECTS) has wide inter-rater variability, and modern studies have also shown that reperfusion therapies can benefit some patients regardless of the ASPECTS. Reproducible imaging metrics that account for the degree of hypo-attenuation on non-contrast computed tomography (NCCT) may be better suited to guide treatments. Here, we evaluate Net Water Uptake (NWU), a novel NCCT metric that can be calculated in a rapid and automated fashion, to determine its predictive performance for identifying clinical outcomes in patients with AIS compared to ASPECTS.

**Methods:**

From our prospectively collected registry encompassing 11 certified stroke centers, we identified patients with AIS. CT images were pre-processed and segmented, then NWU was calculated by automated comparison of density on ipsilateral and contralateral brain regions. Primary outcome was the area under the receiver operating characteristic curve (AUROC) for competing multivariable regression models with Average NWU versus ASPECTS to predict 90-day outcome measured by modified Rankin Scale (mRS). Regression models were adjusted for age, National Institutes of Health Stroke Scale (NIHSS), tPA administration, and endovascular therapy. Secondary analyses included subgroup comparisons of patients with large infarct core and late time window.

**Results:**

Among 402 subjects with anterior circulation AIS, median age was 69 [IQR 57–80], 49.3% were female, median NIHSS was 11 [IQR 5–19], median ASPECTS was 9 [IQR 7–10], and median 90-day mRS was 3 [IQR 1–5]. The ASPECTS-based model performance was not significantly different from the NWU-based model to classify 90-day mRS outcome, with AUROC 0.732 and 0.749, respectively, (*p* = 0.513 with Delong test). Among the subgroups, performance was again similar, including patients with large infarct core (AUROC 0.795 vs. 0.863, *p* = 0.312) and late time window (AUROC 0.638 vs. 0.677, *p* = 0.267).

**Conclusion:**

NWU is a quantitative metric that can be rapidly and automatically obtained from non-contrast CT with comparable performance to ASPECTS when predicting 90-day functional outcome across a wide range of AIS presentations.

## Introduction

Stroke is a leading cause of morbidity and mortality in the United States, and despite the development of thrombolysis and endovascular therapy (EVT) for patients with acute ischemic stroke (AIS), up to half of patients still experience poor clinical outcomes (1–6). Recent randomized trials have shown us that our traditional paradigm to predict who will return to functional independence after AIS is incomplete, and our understanding will now need to go beyond “time is brain” and existing estimates of infarct size on pre-treatment imaging (5–12).

Most stroke centers utilize non-contrast computed tomography (NCCT) to triage patients with ischemic stroke, and a common marker to estimate early infarct severity is the Alberta Stroke Program Early CT Score (ASPECTS) (13, 14). Modern trials have shown us that reperfusion therapies can benefit some patients regardless of how low the ASPECTS is, and there are also a large proportion of patients who do poorly despite having a good ASPECTS (8–10, 15–17). Furthermore, ASPECTS has wide inter-rater variability, and unfortunately in many settings there is limited access to neuroradiology expertise (18, 19). CT perfusion (CTP) imaging has also been used for treatment selection; however, CTP is resource intensive, only effective in a narrow set of circumstances, and can otherwise be plagued with overestimation of the infarct core and inability to identify many infarcts (20–22).

**Figure 1:**
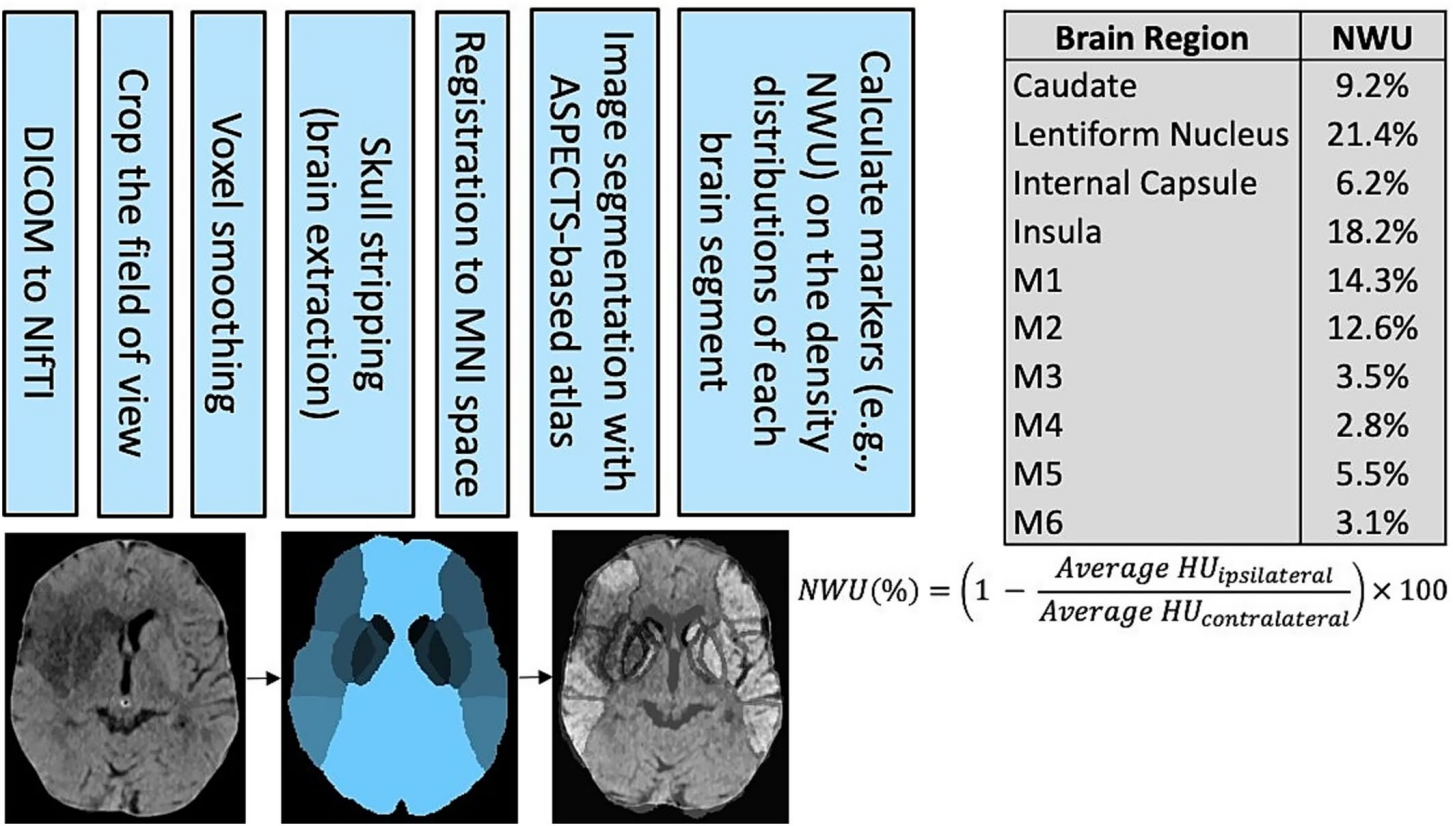
The algorithm for automated net water uptake calculation includes pre-processing, segmentation, and density calculations. On the right, the example results demonstrate how NWU quantifies the degree of injury in each stroke region. DICOM, Digital Imaging and Communications in Medicine; NIfTI, Neuroimaging Informatics Technology Initiative; MNI, Montreal Neurological Institute; ASPECTS, Alberta Stroke Program Early CT Score; NWU, net water uptake; and HU, Hounsfield Units.

Therefore, we aimed in this study to evaluate net water uptake (NWU) as a new NCCT biomarker that could be automated and highly reliable to predict post-stroke outcomes. NWU is a measurement of brain injury and edema based on the degree of hypoattenuation in the stroke area compared to contralateral normal tissue (23). The equation to calculate net water uptake is Net Water Uptake (%) = 1 - (Density_ipsilateral / Density_contralateral) × 100. NWU is a tissue-level measurement and more granular than traditional imaging scores which are evaluated at the subject level. Early studies have shown NWU reliably predicts malignant cerebral edema and poor outcomes after AIS (24–28). Here, we used a prospectively collected registry cohort to evaluate our recently developed automated algorithm that calculates NWU in the primary regions of the anterior circulation territory on NCCT after image registration and segmentation (see Figure 1). We hypothesized that automated NWU will have non-inferior performance compared to neuroradiologist-assessed ASPECTS when predicting post-stroke functional outcomes.

## Methods

### Study cohort

From our prospective registry including 11 certified stroke centers in Houston, TX, USA, we identified consecutive patients who were treated for acute ischemic stroke between 2018 and 2022. All included subjects underwent acute screening in the emergency department with non-contrast CT. The final stroke diagnosis was confirmed clinically and radiographically by a board-certified vascular neurologist, and subjects were excluded if the stroke occurred in the posterior circulation, if the imaging was not interpretable, or if follow-up outcomes were not recorded. The STROBE guidelines were used for the formulation of this study design and manuscript. This study was performed under the guidelines from the Helsinki Declaration and IRB HSC-MS-19-0630 approved by the University of Texas Health Science Center at Houston (UTHealth Houston) IRB and Memorial Hermann Hospital. Data and code will be made available upon reasonable request.

### Clinical measurements

Demographic data and baseline clinical characteristics were recorded, including the use of thrombolysis and endovascular therapy. Imaging characteristics were determined using the radiology reports of the NCCT and CT angiography. Specifically, ASPECTS was determined by expert neuroradiologists and interventional neurologists each with several years of clinical experience. For subjects who underwent EVT, the reperfusion grades were recorded prospectively at the time of the procedure using the thrombolysis in cerebral infarction (TICI) score. The discharge and 90-day clinical outcomes were adjudicated by independent investigators who were not involved in the treatments and were trained in evaluating the modified Rankin Scale and secondary outcomes.

### Imaging analysis

All subjects underwent non-contrast CT at the time of presentation to the emergency department. The acquisition scanners vary between the certified stroke centers and include machines manufactured by GE (LightSpeed, Optima, Discovery, or Revolution), Philips (Ingenuity), Siemens (SOMATOM, Emotion, or Sensation), and Toshiba (Aquilion). All images had a slice thickness of 5 mm, and standardized field of view was applied prior to analysis. All imaging data were de-identified to ensure blinded evaluation. The imaging analysis algorithm developed for this study utilized recommendations from previously validated pre-processing pipelines for CT brain imaging and the steps followed a simple pathway including DICOM to NIfTI format conversion, field of view selection, voxel smoothing, skull stripping (brain extraction), and registration to a standard brain atlas (29–35). The MNI-152 atlas was utilized for this registration (36). The brain images were then segmented into the 10 stroke regions of the anterior circulation using custom image masking (caudate, lentiform nucleus, internal capsule, insula, M1, M2, M3, M4, M5, and M6). Segmentations were visually inspected for accuracy. Voxels were excluded from the density calculation if they were outside the range of 20–50 Hounsfield units, which allowed automatic exclusion of encephalomalacia, calcifications, and acute hemorrhage. Finally, the voxel densities in each region were averaged and the NWU was calculated. In doing so, the output for each NCCT is a list of 10 NWU values corresponding to the 10 standard stroke regions. To derive a single final measurement per subject, two methods of averaging were evaluated: in the primary analysis, a conventional average of all 10 NWU values, and in secondary analysis, a weighted average where the weight is the volume of each region. The conventional average NWU across all 10 regions can reflect both the size of the infarct and the degree of hypoattenuation.

### Study outcomes

The primary outcome was the performance of average NWU and clinical variables to predict 90-day functional outcome measured by the modified Rankin Scale (mRS). Good functional outcome was defined as mRS 0–2, and poor outcome mRS 3–6. The NWU model’s performance was directly compared against a parallel model based on ASPECTS. This 90-day outcome was assessed in secondary subgroups as well, including subjects in the very early time window (0–3 h from last known well), late time window (6–24 h), small presenting infarct core (ASPECTS 6–10), and large infarct core (ASPECTS 0–5). The additional outcomes included the presence of precise neurologic deficits at time of hospital discharge: language impairment, visual impairment, the need for walking assistance, decreased level of consciousness (LOC), arm and leg motor weakness, and severe dysphagia requiring gastrostomy placement. Language impairment was defined as any deficit with fluency or comprehension, and visual impairment was defined as persistent quadrantanopia or hemianopia. Walking assistance was defined as requiring a device for mobility such as a rolling walker or wheelchair. LOC deficit was defined as obtundation or comatose state, and motor weakness was defined as 0 to 3 on the Medical Research Council scale for muscle strength.

### Statistical analysis

Descriptive statistics were used to evaluate the patient demographics and stroke presentation data to understand the baseline characteristics of the entire cohort. The Fisher test for categorical variables and the Wilcoxon Rank Sum test for continuous variables were used to evaluate the differences between patients with favorable and unfavorable primary outcomes. Additionally, imaging characteristics and treatment data were compared for variables such as occlusion location, ASPECTS, tPA administration, and whether or not EVT was performed.

To evaluate the primary outcome, two multivariable logistic regression models were created, adjusting for confounding clinical variables. The first model used the automated average NWU calculation, and the second model used neuroradiologist-assessed ASPECTS. The included confounders were chosen *a priori* because of their known association with post-stroke clinical outcomes, including age, National Institutes of Health Stroke Scale (NIHSS), received tPA, and received EVT. The predictive performance was quantified by the area under the receiver operating characteristic curve (AUROC), and the two models were compared with the Delong test. The cohort was randomly divided 80:20 into training and testing sets to perform this AUROC analysis, and the data partitions maintained the representation of the two outcome classes. To evaluate the secondary outcomes, multivariable logistic regression models were developed and compared in a similar fashion. In addition, to further study the nuanced new biomarker, univariable logistic regression was conducted to determine the association between NWU from specific brain regions and the individual neurologic outcomes. Lastly, we evaluated subsets of the study cohort to explore where NWU and ASPECTS may perform better or worse, including the very early and late time windows as well as small and large infarct cores. For all statistical tests, a *p*-value < 0.05 was considered significant. Analyses were performed with the open-source statistical software R (37).

## Results

Among 402 patients with AIS, median age was 69 [IQR 57–80], 49.3% were female, median NIHSS was 11 [IQR 5–19], and median pre-morbid mRS was 0 [IQR 0–1] (see Table 1). In addition, 67.2% had a large vessel occlusion, median ASPECTS was 9 [IQR 7–10], 44.3% received tPA, 39.1% received endovascular therapy, and median 90-day mRS was 3 [IQR 1–5]. All 402 subjects had successful automated NCCT image processing, and the median time to perform NWU calculations was 87 s [IQR 77–95].

**Table 1:** Baseline and imaging characteristics of patient cohort.

Variable	Total cohort (*n* = 402)	90-day mRS 0–2 (*n* = 180)	90-day mRS 3–6 (*n* = 222)	*p*-value
Age (years), median [IQR]	69 [57, 80]	67 [54, 77]	71 [59, 82]	0.009
Female Sex, *n* (%)	198 (49.3%)	89 (49.4%)	109 (49.1%)	0.51
Race:				
White, *n* (%)	245 (60.9%)	121 (67.2%)	124 (55.9%)	--
Black or African American, *n* (%)	102 (25.4%)	34 (18.9%)	68 (30.6%)	0.007
Asian, *n* (%)	17 (4.2%)	8 (4.4%)	9 (4.1%)	1
Other, *n* (%)	38 (9.5%)	17 (9.4%)	21 (9.5%)	0.61
Ethnicity: Hispanic, *n* (%)	86 (21.4%)	42 (23.3%)	44 (19.8%)	0.39
Diabetes history, *n* (%)	108 (26.9%)	46 (25.6%)	62 (27.9%)	0.50
Hypertension history, *n* (%)	276 (68.7%)	125 (69.4%)	151 (68.0%)	0.91
Hyperlipidemia history, *n* (%)	152 (37.8%)	74 (41.1%)	78 (35.1%)	0.35
Atrial fibrillation history, *n* (%)	65 (16.2%)	27 (15.0%)	38 (17.1%)	0.50
Tobacco use, *n* (%)	78 (19.4%)	46 (25.6%)	32 (14.4%)	0.011
LKW to Arrival (minutes), median [IQR]	280 [103, 670]	202 [98, 510]	407 [106, 738]	0.047
NIHSS on Arrival, median [IQR]	11 [5, 19]	6 [3, 12]	16 [7, 21]	<0.001
Baseline mRS, median [IQR]	0 [0, 1]	0 [0, 1]	0 [0, 2]	<0.001
Occlusion location:				
Intracranial ICA, *n* (%)	59 (14.7%)	20 (11.1%)	39 (17.6%)	--
MCA M1, *n* (%)	132 (32.8%)	44 (24.4%)	88 (39.6%)	1
MCA distal, *n* (%)	61 (15.2%)	29 (16.1%)	32 (14.4%)	0.12
ACA, n (%)	4 (1.0%)	0 (0.0%)	4 (1.8%)	1
No LVO, *n* (%)	132 (32.8%)	84 (46.7%)	48 (21.6%)	<0.001
ASPECTS, median [IQR]	9 [7, 10]	10 [8, 10]	8 [5, 10]	<0.001
CTP infarct core estimation (mL), mean +/− SD	27.1 +/− 45.4	4.8 +/− 15.4	18.3 +/− 41.8	0.007
Received IV tPA, *n* (%)	178 (44.3%)	90 (50.0%)	88 (39.6%)	0.043
Received Endovascular Therapy, *n* (%)	157 (39.1%)	57 (31.7%)	100 (45.0%)	0.007
Endovascular outcome TICI 2b-3, *n* (% of those who received EVT)	139 (88.5%)	55 (96.5%)	84 (84.0%)	0.052
Length of Stay (days), median [IQR]	4 [2, 7]	3 [2, 5]	5 [3, 9]	<0.001
90-day mRS, median [IQR]	3 [1, 5]	1 [0, 1]	4 [4, 6]	<0.001

IQR, interquartile range; LKW, last known well; NIHSS, National Institutes of Health Stroke Scale; mRS, modified Rankin Scale; ICA, internal carotid artery; MCA, middle cerebral artery; ACA, anterior cerebral artery; LVO, large vessel occlusion; ASPECTS, Alberta Stroke Program Early CT Score; CTP, CT Perfusion, mL, milliliters; tPA, tissue plasminogen activator; TICI = thrombolysis in cerebral infarction; EVT, endovascular therapy. Continuous variables were compared using Wilcoxon rank sum test, and categorical variables were compared using Fisher’s exact test. All analyses were performed in the R statistical software.

**Figure 2:**
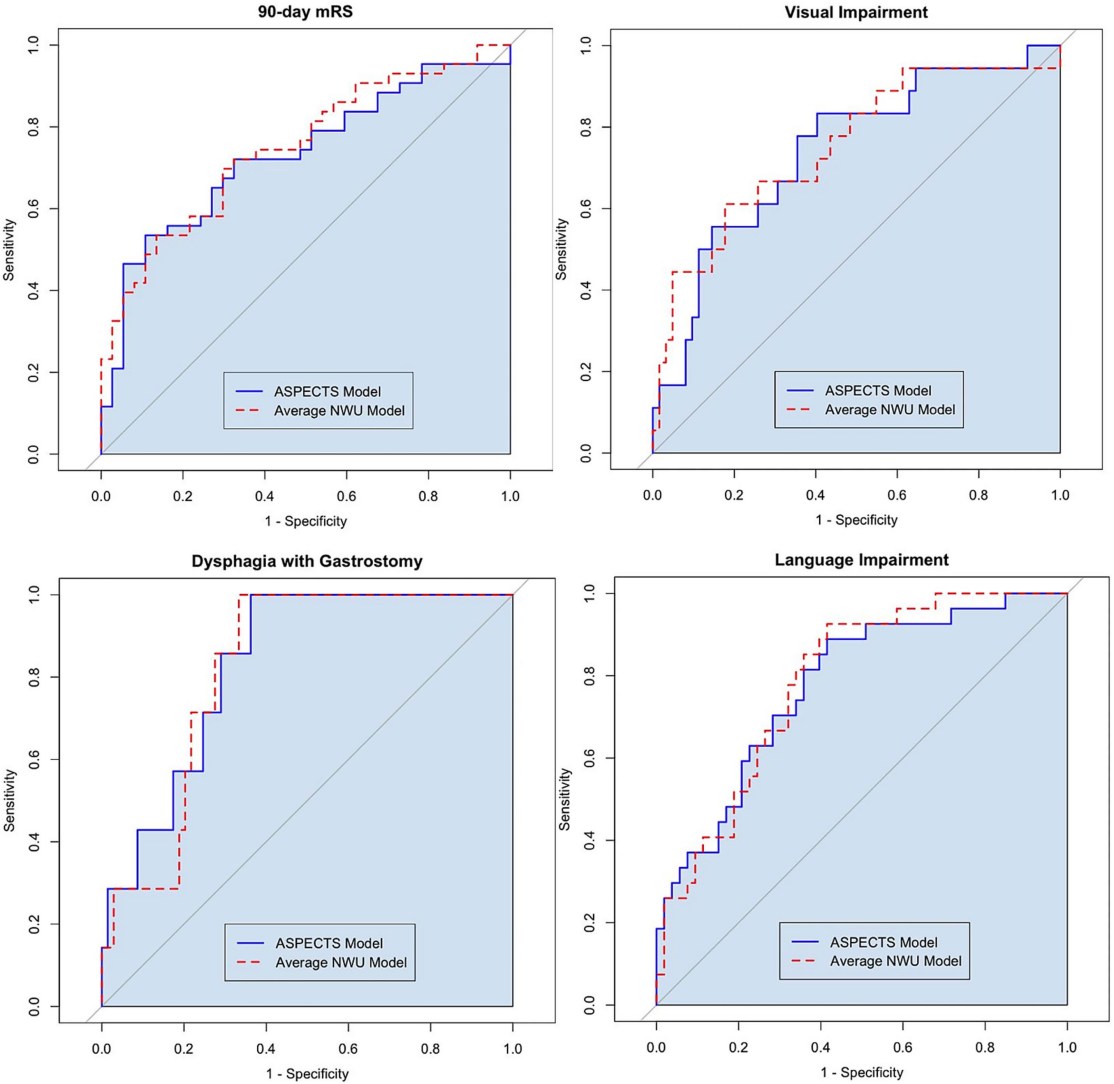
To evaluate the predictive performance of the ASPECTS and NWU models, the AUROC values were compared with the Delong test. When predicting 90-day mRS 3–6, AUROC 0.732 for the ASPECTS-based model vs. AUROC 0.749 for the NWU-based model (*p* = 0.513). When predicting visual Impairment, AUROC 0.743 for the ASPECTS-based model vs. AUROC 0.752 for the NWU-based model (*p* = 0.773). When predicting dysphagia with gastrostomy, AUROC 0.832 for the ASPECTS-based model vs. AUROC 0.822 for the NWU-based model (*p* = 0.724). When predicting language Impairment, AUROC 0.776 for the ASPECTS-based model vs. AUROC 0.787 for the NWU-based model (*p* = 0.639).

In multivariable logistic regression, lower ASPECTS and higher average NWU was associated with greater likelihood of poor functional outcome measured by 90-day mRS (OR 0.84 [CI 0.74, 0.95] and OR 1.14 [CI 1.02, 1.26], respectively). See Table 2 for full results. In ROC analysis, the ASPECTS-based model performed the same as the NWU-based model when classifying 90-day mRS outcome, with AUROC 0.732 and 0.749, respectively, (*p* = 0.513 with Delong test, see Figure 2).

**Table 2:** Multivariable regression analysis to predict poor 90-day clinical outcome (mRS 3–6) with ASPECTS-based model and NWU-based model.

Variable	Odds ratio	95% Confidence interval	*p*-value
ASPECTS-based Model			
Age (years)	1.02	[1.00, 1.03]	0.017
NIHSS on arrival	1.10	[1.06, 1.14]	<0.001
Received IV tPA	0.48	[0.31, 0.76]	0.002
Received Endovascular Therapy	0.71	[0.42, 1.21]	0.21
ASPECTS	0.84	[0.74, 0.95]	0.008
NWU-based Model			
Age (years)	1.02	[1.00, 1.03]	0.046
NIHSS on arrival	1.11	[1.07, 1.15]	<0.001
Received IV tPA	0.50	[0.31, 0.78]	0.003
Received Endovascular Therapy	0.79	[0.46, 1.33]	0.37
Average NWU	1.14	[1.02, 1.26]	0.019

ASPECTS, Alberta Stroke Program Early CT Score, NIHSS, National Institutes of Health Stroke Scale, tPA, tissue plasminogen activator, NWU, net water uptake. All analyses were performed in the R statistical software.

In the secondary analysis, the ASPECTS and NWU models showed varying levels of performance to predict 90-day mRS outcome among different clinically relevant subgroups (see Table 3). The NWU-based model had excellent performance when classifying 90-day mRS outcome for patients with large infarct core at presentation defined as ASPECTS 0–5 (AUROC 0.863). In addition, the models seemed to perform better for the subgroup presenting in the very early time window (less than 3 h from last known well) compared to the late time window. Overall, the differences in performance between subgroups that were seen by the ASPECTS-based model were mirrored by the NWU-based model.

**Table 3:** Exploring the performance of NWU and ASPECTS in clinically relevant subgroups to predict poor 90-day functional outcome.

Patient subset	ASPECTS model performance (AUROC)	NWU model performance (AUROC)	*p*-value
Large vessel occlusion, *n* = 270	0.775	0.770	0.76
Very early time window (presenting less than 3 h from last known well), *n* = 86	0.734	0.743	0.34
Late time window (presenting 6 to 24 h from last known well), *n* = 100	0.638	0.677	0.27
Small estimated infarct core at presentation (ASPECTS 6–10), *n* = 336	0.713	0.715	0.76
Large estimated infarct core at presentation (ASPECTS 0–5), *n* = 66	0.795	0.863	0.31

Both models included the confounder variables age, NIHSS, received tPA, and received EVT. Multivariable logistic regression models were developed to predict poor 90-day mRS 3–6, and AUROC values were statistically compared using the Delong test. All analyses were performed in the R statistical software. ASPECTS, Alberta Stroke Program Early CT Score; NWU, net water uptake; AUROC, area under the receiver operating characteristic curve; NIHSS, National Institutes of Health Stroke Scale; tPA, tissue plasminogen activator; EVT, endovascular therapy; mRS, modified Rankin Scale.

Among the secondary outcomes, average NWU showed a significant association in multivariable logistic regression with several individual neurologic deficits including language impairment, visual impairment, severe dysphagia, arm and leg motor weakness, and LOC deficit at hospital discharge (see Supplemental Table 1). The NWU and ASPECTS-based models performed similarly, and this performance was consistently excellent or very good based on AUROC (see Figure 2 and Supplementary Figure 1). For example, when predicting language impairment at discharge, the NWU-based model showed an AUROC of 0.787 versus the ASPECTS-based model AUROC of 0.776 (*p* = 0.639 with Delong test).

**Figure 3:**
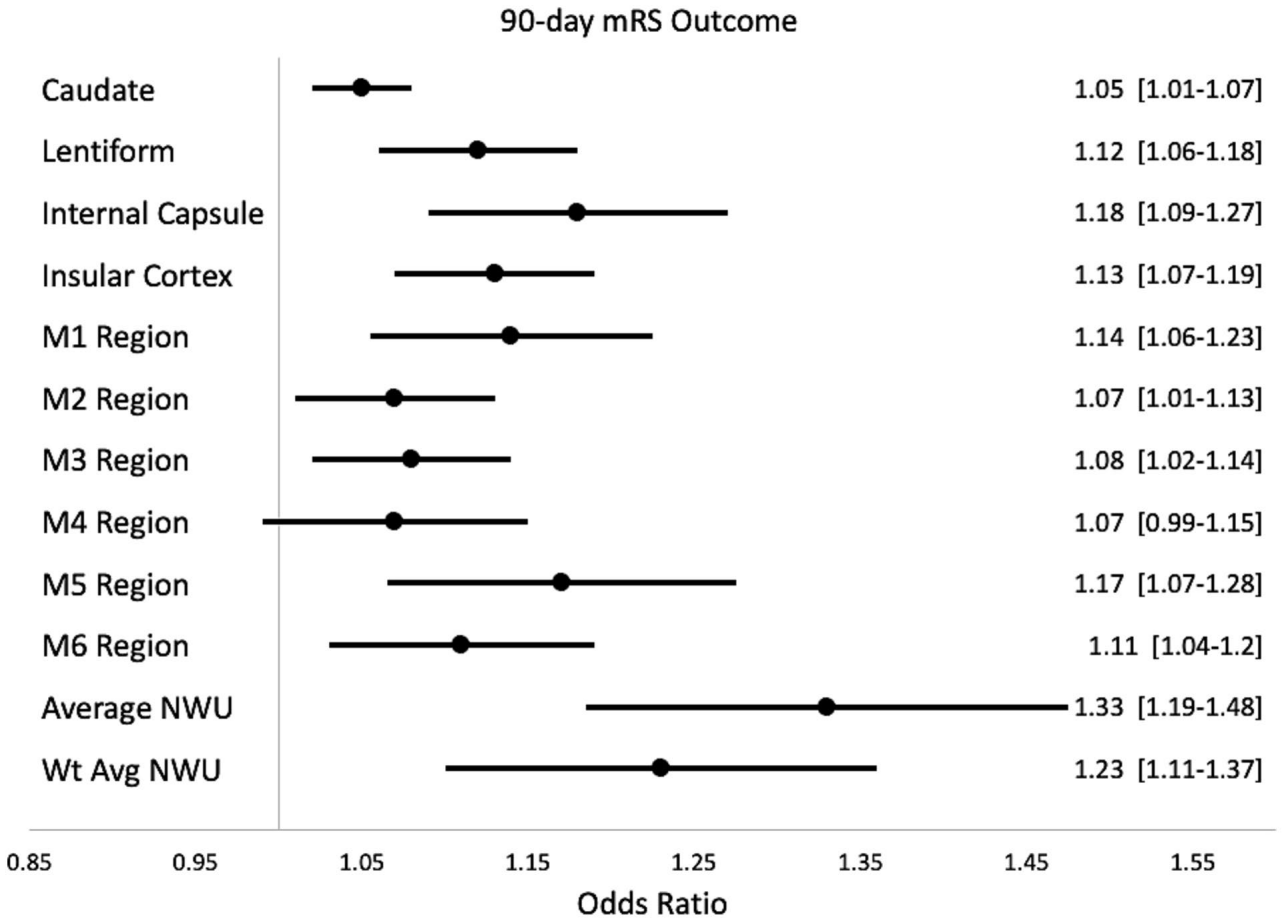
The secondary analysis evaluated the association of NWU from individual regions and 90-day Functional Outcome. The 10 stroke regions are represented as well as Average NWU and Weighted Average NWU. On the right, the results of univariable logistic regression are displayed for the prediction of 90-day mRS outcome (represented with Odds Ratio and 95% confidence intervals).

**Figure 4:**
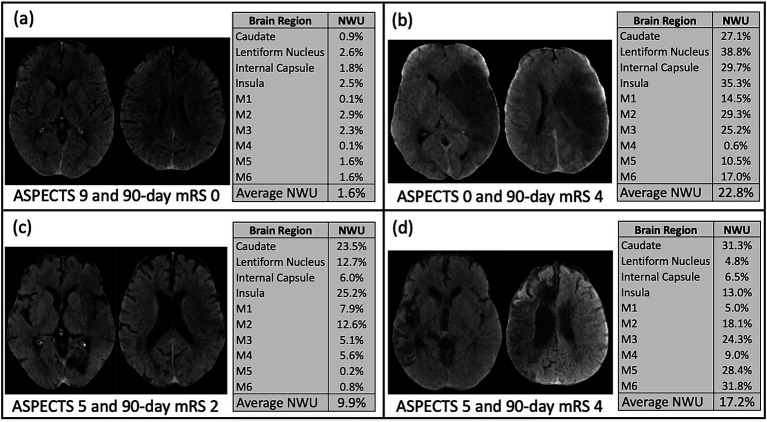
These four examples from the study cohort demonstrate common clinical scenarios. **(a)** Subject with ASPECTS 9, average NWU 1.6%, and 90-day mRS 0; **(b)** Subject with ASPECTS 0, average NWU 22.8%, and 90-day mRS 4; **(c)** Subject with ASPECTS 5, average NWU 9.9%, and 90-day mRS 2; **(d)** Subject with ASPECTS 5, average NWU 17.2%, and 90-day mRS 4.

When examining the association between individual brain regions and specific neurologic deficits, logistic regression showed that most of the brain regions showed consistently significant predictive power for the precise deficits except for the caudate and M4 region (see Figure 3 and Supplemental Figure 2). Particular deficits were not isolated to certain brain regions, but rather NWU in almost any region showed significant association to each precise outcome. Lastly, four case examples are shown in Figure 4 to demonstrate some common clinical scenarios and the resulting ASPECTS and NWU findings.

## Discussion

In this cohort study of over 400 patients with anterior circulation acute ischemic stroke, we demonstrate the utility of a novel imaging biomarker, net water uptake, that quantifies hypoattenuation in brain regions on NCCT in a fully automated manner. We found that the performance of this marker, which can be calculated within 2 min, is equivalent to expert neuroradiologist-defined ASPECT scoring, across the entire cohort as well as select crucial subgroups.

One of the first clinical studies of NWU investigated its use as a “tissue-clock” to predict ischemic stroke time window based on non-contrast CT (23). Studies have also demonstrated that NWU is useful for predicting midline shift and the presence of malignant cerebral edema to potentially inform the need for decompressive hemicraniectomy (24, 27, 28). Some of these early studies have relied on advanced imaging for segmentation of infarct core prior to calculating NWU in the predicted core or region-of-interest, so we sought to develop an agnostic approach that calculated NWU in 10 standard regions using NCCT alone (23, 24, 27, 28, 38). In addition, some studies have relied on commercial software from biotechnology companies which limits widespread use compared to this open-access approach (26–28). Lastly, instead of predicting imaging outcomes, our study focused on the prediction of long-term clinical outcomes that can help guide treatment decision-making and patient expectations. This study demonstrated that automated NWU is highly reliable as an independent predictor of these clinical outcomes and performs equally to the ASPECT score which requires a subjective visual assessment by a trained neuroradiologist or neurologist. NWU provides a granular measurement of tissue injury and edema which adds new quantitative information beyond the 0 to 10 scale of the ASPECTS. We suspect this imaging marker is quantifying localized edema from irreversible ischemia and early immunological response (38–41). Not only could NWU be studied in future trials to improve treatment selection for thrombolysis and EVT, but it could also prove to be useful to stratify patients for new investigative treatments such as neuroprotectants and immunomodulatory agents.

In the secondary analysis, we observed that NWU and ASPECTS generally have a stronger association with 90-day outcome for patients in the very early time window and also those who are presenting with a large infarct core. This study was not powered to identify differences in biomarker performance among these subpopulations, so future studies will be focused on evaluating the utility of NWU to triage these difficult cases. For example, recent randomized controlled trials showed a benefit of endovascular therapy for patients with ASPECTS in the range of 0 to 5, suggesting the scale can no longer be relied upon for EVT decision-making (8–10). Automated NWU could not only fill this gap, but it could be accomplished with freely available software and only non-contrast CT.

Clinical scales like the mRS and the NIHSS are not always reflective of how patients rate their own disability after stroke, are biased toward motor disability, and are not fully reliable to gage long-term disability (42–47). We anticipate that a more personalized approach is required in the future of stroke care, and in this study NWU has also shown strong association with individual neurologic outcomes that impact a patient’s quality of life and daily activities. The NWU from individual regions of the brain were shown to have a strong association with precise neurologic deficits such as language impairment, visual impairment, and severe dysphagia. Furthermore, these secondary outcomes were assessed at time of hospital discharge instead of long-term follow up, because 90-day outcomes are influenced by many factors that are not directly stroke related including insurance status, resource availability, and systemic disparities. For example, recent studies have demonstrated that lower rates of acute and post-acute treatments were observed in Black patients with stroke compared to their White counterparts (48–51). Our study demonstrated a similar finding in that there was a significant difference in rate of good 90-day mRS among Non-Hispanic Black patients compared to White patients (Table 1), but there was no significant difference in disability between races at the time of hospital discharge (26% vs. 20% respectively, *p* = 0.117). Although this study was not focused on this research question, the finding suggests that there may be disparities in post-hospital stroke care that affect long-term recovery and should be further investigated in future studies.

This study has limitations. Because of the observational and retrospective nature of this cohort study, the lack of randomization can introduce confounding factors that are not fully accounted for in the baseline comparison in Table 1. The primary analysis did not include all possible co-variables, so there is potential for residual confounding. On the other hand, the population is representative of commonly seen cohorts with AIS in both real-world practice and prospective randomized trials (1, 4, 7). Also, the study cohort only sampled from a single geographic region, a large metropolitan area in the southern United States (Houston, TX), so the findings will need to be replicated in a geographically diverse future study. Additionally, the study population contained both patients with and without LVO, yet the novel imaging marker was still strongly associated with the outcomes in subgroups. As an automated and easy-to-use NCCT triage tool, NWU could be widely applicable among patients with AIS, however future trials can investigate its potential benefit for treatment decision-making among sub-populations and for specific reperfusion or neuroprotectant therapies. When compared to ASPECTS, automated NWU is invulnerable to subjectivity and inter-rater variability, provides a new degree of nuance to NCCT evaluation, and could be used even in settings where vascular neurology and neuroradiology expertise are not available.

In summary, we found that a fully automated NWU assessment provided quantitative evaluation of ischemia equivalent to expert neuroradiologist-assessed ASPECT scoring when predicting clinical outcomes. Because of its ease of acquisition and quantified outputs that are not subject to inter-rater disagreements, NWU may serve as a useful tool for clinical practice and upcoming clinical trials.

## Data Availability

The raw data supporting the conclusions of this article will be made available by the authors, without undue reservation.
