# Supplementary material for: A novel automated CT biomarker to predict outcomes in acute ischemic stroke: net water uptake
Source: Front Neurol. 2025 Aug 22;16:1629434. doi: 10.3389/fneur.2025.1629434 (PMC12411213; doi:10.3389/fneur.2025.1629434)

**Supplemental Material**

**Supplemental Table 1. Multivariable Logistic Regression to Predict Secondary Outcomes with ASPECTS-based Model and NWU-based Model**

| **Variable** | **Language Deficit**  **OR and 95% CI** | **Language Deficit**  **p-value** | **Visual Impairment**  **OR and 95% CI** | **Visual Impairment p-value** | **Walking Assistance OR and**  **95% CI** | **Walking Assistance**  **p-value** | **Dysphagia with Gastrostomy**  **OR and 95% CI** | **Dysphagia**  **p-value** | **Arm Weakness OR and 95% CI** | **Arm Weakness p-value** | **Leg Weakness OR and 95% CI** | **Leg Weakness p-value** | **LOC Deficit OR and 95% CI** | **LOC Deficit p-value** |
| --- | --- | --- | --- | --- | --- | --- | --- | --- | --- | --- | --- | --- | --- | --- |
|  | | | | | | | | | | | | | | |
| Age (years) | 1.02 [0.99,1.03] | 0.066 | 1.01 [0.99,1.03] | 0.126 | 1.03 [1.01,1.05] | **0.001** | 1.00 [0.98,1.02] | 0.941 | 1.01 [0.99,1.03] | 0.176 | 1.01 [0.99,1.03] | 0.153 | 1.03 [1.01,1.05] | **0.012** |
| NIHSS on arrival | 1.15 [1.10,1.19] | **<0.001** | 1.10 [1.06,1.14] | **<0.001** | 1.14 [1.08,1.20] | **<0.001** | 1.16 [1.10,1.22] | **<0.001** | 1.16 [1.11,1.21] | **<0.001** | 1.14 [1.10,1.19] | **<0.001** | 1.17 [1.12,1.22] | **<0.001** |
| Received IV tPA | 0.61 [0.37,0.99] | **0.050** | 0.51 [0.30,0.84] | **0.009** | 0.53 [0.31,0.90] | **0.019** | 0.70 [0.35,1.43] | 0.330 | 0.51 [0.30,0.86] | **0.011** | 0.58 [0.35,0.98] | **0.040** | 0.39 [0.21,0.73] | **0.003** |
| Received EVT | 0.72 [0.41,1.25] | 0.241 | 0.49 [0.28,0.85] | **0.011** | 0.94 [0.47,1.89] | 0.872 | 0.81 [0.38,1.71] | 0.580 | 0.55 [0.31,0.99] | **0.047** | 0.52 [0.29,0.93] | **0.028** | 0.37 [0.20,0.70] | **0.002** |
| ASPECTS | 0.79 [0.69,0.90] | **0.001** | 0.79 [0.70,0.89] | **<0.001** | 0.87 [0.73,1.05] | 0.140 | 0.79 [0.68,0.93] | **0.005** | 0.75 [0.65,0.86] | **<0.001** | 0.73 [0.64,0.84] | **<0.001** | 0.82 [0.72,0.93] | **0.002** |
|  | | | | | | | | | | | | | | |
| Age (years) | 1.01 [0.99,1.03] | 0.183 | 1.01 [0.99,1.03] | 0.309 | 1.03 [1.01,1.05] | 0.003 | 0.99 [0.97,1.01] | 0.493 | 1.01 [0.99,1.02] | 0.452 | 1.01 [0.99,1.02] | 0.459 | 1.02 [1.00,1.04] | **0.031** |
| NIHSS on arrival | 1.16 [1.12,1.21] | **<0.001** | 1.12 [1.08,1.16] | **<0.001** | 1.14 [1.08,1.20] | **<0.001** | 1.17 [1.11,1.23] | **<0.001** | 1.18 [1.13,1.23] | **<0.001** | 1.16 [1.12,1.21] | **<0.001** | 1.18 [1.13,1.23] | **<0.001** |
| Received IV tPA | 0.63 [0.38,1.03] | 0.065 | 0.52 [0.31,0.86] | **0.011** | 0.55 [0.32,0.94] | **0.029** | 0.72 [0.36,1.48] | 0.378 | 0.52 [0.31,0.88] | **0.015** | 0.61 [0.37,1.01] | 0.054 | 0.39 [0.21,0.74] | **0.004** |
| Received EVT | 0.81 [0.47,1.41] | 0.465 | 0.55 [0.32,0.95] | **0.032** | 1.03 [0.52,2.05] | 0.938 | 1.02 [0.48,2.16] | 0.967 | 0.64 [0.36,1.14] | 0.133 | 0.62 [0.35,1.10] | 0.100 | 0.40 [0.21,0.77] | **0.006** |
| Average NWU | 1.18 [1.05,1.32] | **0.004** | 1.17 [1.06,1.29] | **0.002** | 1.15 [0.99,1.34] | 0.074 | 1.15 [1.03,1.28] | **0.015** | 1.20 [1.07,1.35] | **0.002** | 1.23 [1.09,1.38] | **0.001** | 1.12 [1.02,1.24] | **0.018** |
| OR = Odds ratio, CI = confidence interval, LOC = level of consciousness, ASPECTS = Alberta Stroke Program Early CT Score, NIHSS = National Institutes of Health Stroke Scale, tPA = tissue plasminogen activator, EVT = endovascular therapy, NWU = net water uptake. All analyses were performed in the R statistical software. | | | | | | | | | | | | | | |

**Supplemental Figure 1.** The predictive performance of the ASPECTS and NWU models were analyzed for the other secondary outcomes and the AUROC values were compared with the Delong test. Multivariable logistic regression was again utilized with confounder variables age, NIHSS, received tPA, and received EVT. When predicting decreased level of consciousness, AUROC 0.797 for the ASPECTS-based model vs. AUROC 0.797 for the NWU-based model (p = 1). When predicting arm motor weakness, AUROC 0.853 for the ASPECTS-based model vs. AUROC 0.833 for the NWU-based model (p = 0.422). When predicting leg motor weakness, AUROC 0.843 for the ASPECTS-based model vs. AUROC 0.808 for the NWU-based model (p = 0.206). When predicting walking assistance requirement, AUROC 0.795 for the ASPECTS-based model vs. AUROC 0.768 for the NWU-based model (p = 0.396).


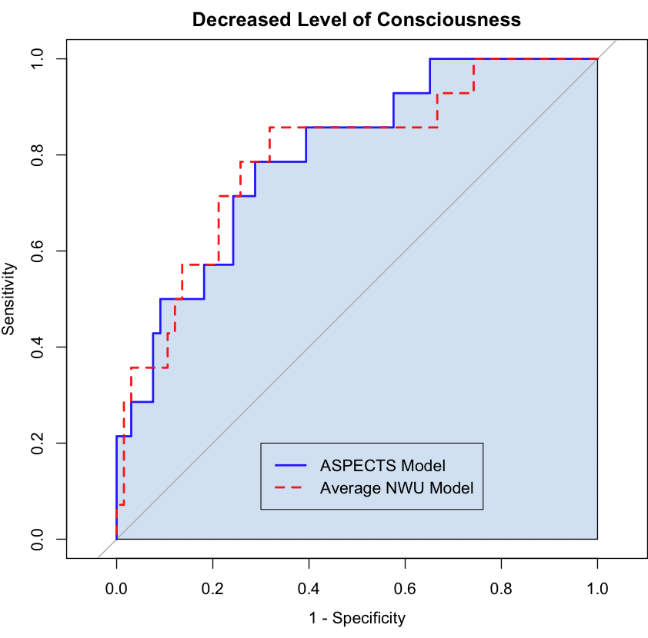

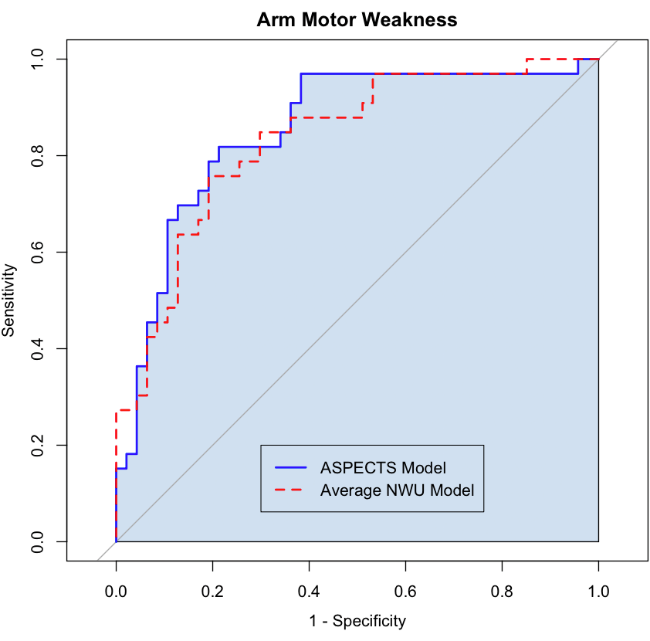


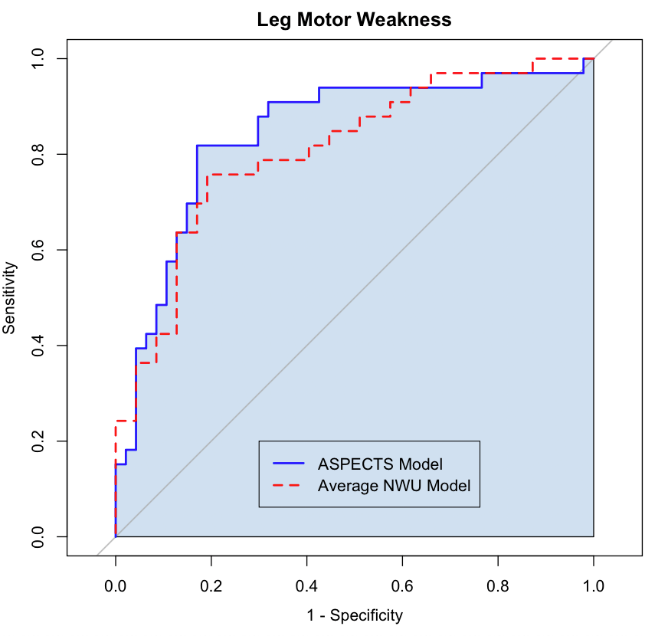

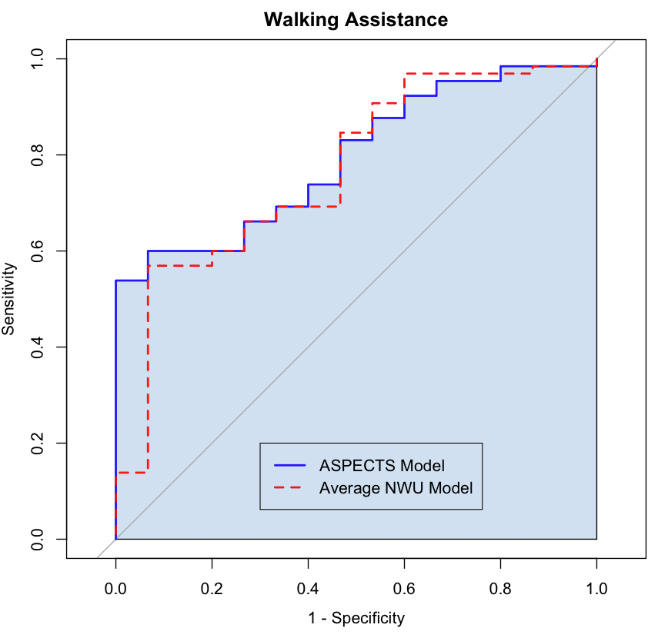


**Supplemental Figure 2.** Association between NWU of individual regions and precise neurologic outcomes after acute ischemic stroke. On the left, the ten stroke regions are represented as well as Average NWU and Weighted Average NWU. On the right, the results of univariable logistic regression are represented with Odds Ratio and 95% confidence intervals.


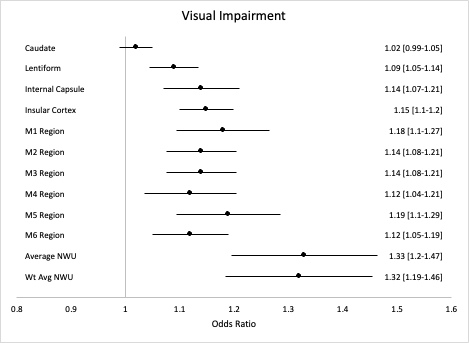

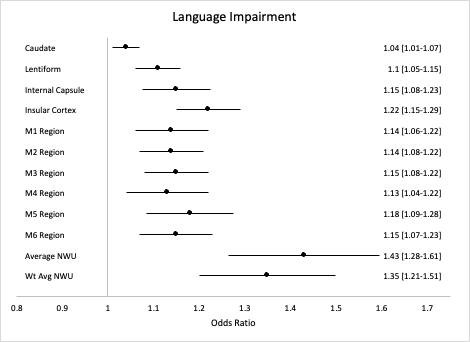


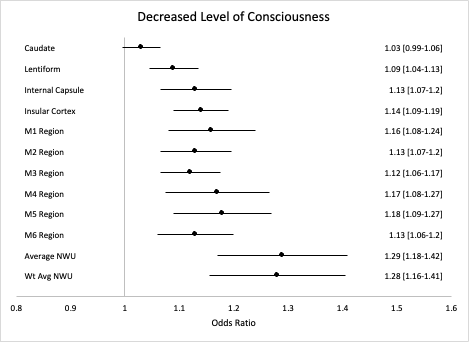

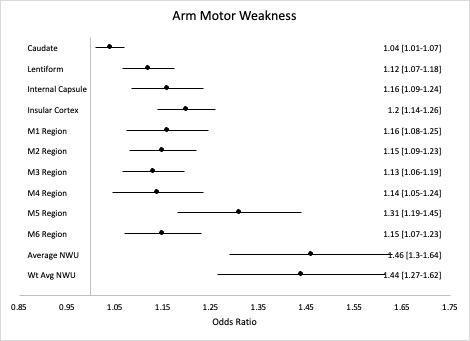

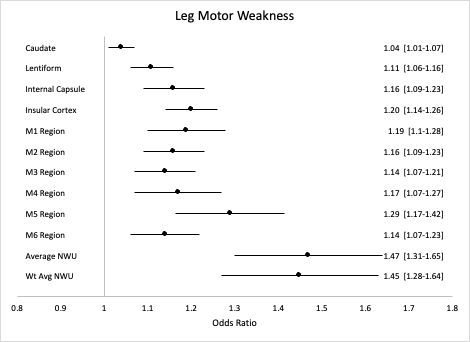

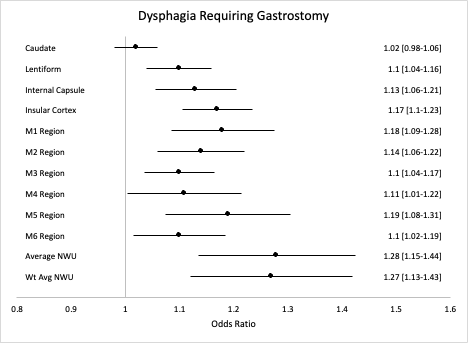

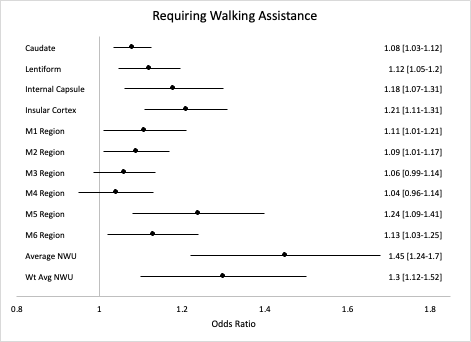

Supplement: Supplementary file 1 [file Table_1.docx]
